# Ceramides are decreased after liraglutide treatment in people with type 2 diabetes: a post hoc analysis of two randomized clinical trials

**DOI:** 10.1186/s12944-023-01922-z

**Published:** 2023-09-26

**Authors:** Asger Wretlind, Viktor Rotbain Curovic, Andressa de Zawadzki, Tommi Suvitaival, Jin Xu, Emilie Hein Zobel, Bernt Johan von Scholten, Rasmus Sejersten Ripa, Andreas Kjaer, Tine Willum Hansen, Tina Vilsbøll, Henrik Vestergaard, Peter Rossing, Cristina Legido-Quigley

**Affiliations:** 1 Steno Diabetes Center Copenhagen, Herlev, Denmark; 2 Department of Clinical Medicine, Faculty of Health and Medical Sciences, University of Copenhagen, Copenhagen, Denmark; 3 King’s College London, London, UK; 4 Novo Nordisk A/S, Måløv, Denmark; 5 Department of Clinical Physiology and Nuclear Medicine & Cluster for Molecular Imaging, Copenhagen University Hospital – Rigshospitalet & Department of Biomedical Sciences, University of Copenhagen, Copenhagen, Denmark; 6 Department of Medicine, Bornholms Hospital, Rønne, Denmark

**Keywords:** Cardiovascular disease, Ceramide, Liraglutide, Type 2 diabetes

## Abstract

**Background:**

Specific ceramides have been identified as risk markers for cardiovascular disease (CVD) years before onset of disease. Treatment with the glucagon-like peptide-1 receptor agonist (GLP-1RA) liraglutide has been shown to induce beneficial changes in the lipid profile and reduce the risk of CVD. Reducing lipotoxic lipids with an antidiabetic drug therapy could be a path towards precision medicine approaches for the treatment of complications to diabetes. In this *post-hoc* study, an investigation was carried out on the effect of liraglutide on CVD-risk associated ceramides in two randomized clinical trials including participants with type 2 diabetes (T2D).

**Methods:**

This study analyzed plasma samples from two independent randomized placebo-controlled clinical trials. The first trial, Antiproteinuric Effects of Liraglutide Treatment (LirAlbu12) followed a crossover design where 27 participants were treated for 12 weeks with either liraglutide (1.8 mg/d) or placebo, followed by a four-week washout period, and then another 12 weeks of the other treatment. The second clinical trial, Effect of Liraglutide on Vascular Inflammation in Type-2 Diabetes (LiraFlame26), lasted for 26 weeks and followed a parallel design, where 102 participants were randomized 1:1 to either liraglutide or placebo. Heresix prespecified plasma ceramides were measured using liquid chromatography mass spectrometry and assessed their changes using linear mixed models. Possible confounders were assessed with mediation analyses.

**Results:**

In the LiraFlame26 trial, 26-week treatment with liraglutide resulted in a significant reduction of two ceramides associated with CVD risk, C16 Cer and C24:1 Cer (*p* < 0.05) compared to placebo. None of the remaining ceramides showed statistically significant changes in response to liraglutide treatment compared to placebo. Significant changes in ceramides were not found after 12-weeks of liraglutide treatment in the LirAlbu12 trial. Mediation analyses showed that weight loss did not affect ceramide reduction.

**Conclusions:**

It was demonstrated that treatment with liraglutide resulted in a reduction in C16 Cer and C24:1 Cer after 26 weeks of treatment. These findings suggest the GLP-1RA can be used to modulate ceramides in addition to its other properties.

**Trial registration:**

Clinicaltrial.gov identifier: NCT02545738 and NCT03449654.

**Supplementary Information:**

The online version contains supplementary material available at 10.1186/s12944-023-01922-z.

## Background

Type 2 diabetes (T2D) is a chronic disease that can lead to comorbidities and excess mortality. The risk of cardiovascular (CV) disease (CVD) is increased with T2D, making CVD the leading cause of death among individuals with T2D [1, 2]. Ceramides are a class of lipids associated with lipotoxic signaling, inflammation and apoptosis [3]. They are present in the circulation as part of low-density lipoprotein (LDL) and very low-density lipoprotein (VLDL) particles [4]. Recent studies have identified a set of specific ceramides that are strongly associated with the risk of CVD [5–9]. Specifically, Ceramide(d18:1/16:0), Ceramide(d18:1/18:0), Ceramide(d18:1/20:0), Ceramide(d18:1/22:0), Ceramide(d18:1/24:0) and Ceramide(d18:1/24:1), which will be referred to in their abbreviated names as C16 Cer, C18 Cer, C20 Cer, C22 Cer, C24 Cer, and C24:1 Cer, respectively. C16 Cer, C18 cer, and C24:1 Cer are associated with increased CVD risk [6, 9], while C22 Cer and C24 Cer are associated with decreased CVD risk [7]. C20 Cer and C22 Cer appear to be weaker CVD predictors as there have been conflicting reports [5, 8].

Individuals with T2D typically have elevated levels of C16 Cer, C18 Cer, C20 Cer and C24:1 Cer [10–12]. C16 Cer, C18 Cer, C20 Cer, C22 Cer and C24 Cer have been associated with insulin resistance [13–15] and C18 Cer, C20 Cer and C22 Cer have been linked to the risk of developing T2D [16–19]. Improvement in insulin sensitivity following gastric bypass surgery has been correlated with reduction in C18 Cer and C22 Cer levels, and low baseline levels of these ceramides were found to predict a higher likelihood of diabetes remission [20, 21]. Studies in mice have shown that reducing these ceramides can reduce insulin resistance [22–24] and development of CVD [25, 26].

We previously demonstrated, in the trial called Effect of Liraglutide on Vascular Inflammation in Type-2 Diabetes (LiraFlame26), that treatment with the glucagon-like peptide-1 receptor agonist (GLP-1RA) liraglutide (1.8 mg/d) downregulated several lipid species, including ceramides in individuals with T2D, using an untargeted lipidomic approach [27]. Conversely, GLP-1RAs, including liraglutide, have been shown to reduce the risk of CV outcomes [28–31]. A better understanding of liraglutide’s effect on ceramide dynamics could provide insight into the observed cardioprotective effects of GLP-1RAs. By reducing lipotoxic molecules, drug therapies for diabetes could facilitate managing diabetes and its complications. In this study, a post-hoc analysis were conducted of plasma ceramides from two randomized, double-blinded, placebo-controlled trials involving 27 and 102 individuals with T2D, respectively, to investigate the effect of liraglutide treatment on the CVD biomarkers C16 Cer, C18 Cer, C20 Cer, C22 Cer, C24 Cer, and C24:1 Cer.

## Methods

### Participants and study design

Plasma samples for this study were acquired from participants in two randomized clinical trials. The first trial, named Antiproteinuric Effects of Liraglutide Treatment (LirAlbu12), was a randomized double-blinded crossover clinical trial conducted at Steno Diabetes Center Copenhagen between 2015 and 2016. The trial, which aimed at studying the effect of liraglutide on albuminuria in T2D, has previously been described in detail [32]. In brief, the trial included participants with T2D who had persistent albuminuria (≥ 30 mg/g in at least 2 of 3 urine samples at inclusion) and who received renin-angiotensin-system blocking therapy. The participants were randomly assigned to receive either 12 weeks of subcutaneous liraglutide (up to 1.8 mg/day) or placebo, in addition to standard care. After completing the first treatment regimen, the participants underwent a 4-week washout period before crossing over to the opposite treatment for the next 12 weeks. Plasma samples were collected before and after each treatment regimen and were analyzed for the 27 participants who completed the trial. The trial is registered at Clinicaltrial.gov under the identifier NCT02545738.

The second study included is a randomized double-blinded parallel clinical trial here called LiraFlame26. LiraFlame26 was carried out to evaluate anti-atherogenic effects of liraglutide in T2D, the protocol has previously been published [33]. The trial was conducted at Steno Diabetes Center Copenhagen between 2017 and 2019 and recruited 102 participants with T2D who were randomized to either subcutaneous liraglutide (up to 1.8 mg/day) or placebo for 26 weeks. Plasma samples were collected at baseline and end of trial. LiraFlame26 was registered at Clinicaltrial.gov with the identifier NCT03449654.

Both trials were approved by regional ethics committee and the Danish Medicine Agency and followed the principles laid out by the Declaration of Helsinki and Good Clinical Practice. Participants provided written informed consent before their enrollment in the study.

### Lipid analyses

Lipids were extracted from the plasma samples obtained from the LirAlbu12 trial using a modified Folch procedure previously reported [34, 35]. The samples were prepared in random order before being analyzed, and pooled samples and blanks were included between every 12 samples for quality control. A set calibration curve samples were analyzed at the start and in the end of the analytical run, using a serial dilution ranging between 0.25 – 200 µg/ml of the pure reference standards C16 Cer, C18 Cer and C24 Cer. The extracted samples were analyzed using an Infinity II ultra-high-performance liquid chromatography system coupled with an Agilent 6550 quadrupole time-of-flight mass spectrometry from Agilent Technologies.

The raw mass spectrometry data from a previously published work of LiraFlame26 [27] were reanalyzed for this work, in parallel with and in the same manner as LirAlbu12. Preprocessing of the mass spectrometry data was carried out using MZmine2 v.2.28 [36], targeting the six ceramides of interest in positive ionization mode: C16 Cer, C18 Cer, C20 Cer, C22 Cer, C24 Cer and C24:1 Cer. The ceramides produced a water loss adduct [M-H2O + H]^+^, used as the quantifier ion, and a protonated adduct [M + H]^+^, used as a qualifier ion [37]. The ceramide peaks in LirAlbu12 were normalized to a pure exogenous internal standard, Ceramide(d18:1/17:0) (C17 Cer), which was spiked into all samples at the same concentration. Calibration curves were constructed by linearly fitting the peak areas of the calibration curve standards, normalized by the internal standard, against their respective concentrations. Absolute quantification in the LirAlbu12 trial was carried out by fitting each ceramide to the closest standard curve. For C20 Cer, this meant fitting to the C18 Cer standard curve, while the C24 Cer standard curve was used to calculate the concentrations of C22 Cer and C24:1 Cer. In contrast, no calibration curves were available for the LiraFlame26 trial, “amount” signifies ceramide peak area normalized to the exogenous standard, C17 Cer, absolute concentrations were not obtained. Outliers, defined as measures more than 3 standard deviations away from the median, were truncated.

### Statistical analyses

Baseline characteristics, presented as mean (SD) or n (%), were compared between the liraglutide and placebo groups using the chi-square test for categorical variables and ANOVA for continuous variables. These results were compiled into a table using the tableone package in R [38]. Nominal *P*-values were kept. To assess the changes in ceramide levels after each treatment, linear mixed models were built for each ceramide level as a function of treatment and time considering random effects between participants [27], using the lme4, lmerTest and ggeffects packages [39–41]. Changes were visualized with boxplots depicting ceramide amount before and after each treatment regimen using the ggplot2 and ggpubr packages [42, 43]. The ceramide changes from the linear mixed models were not adjusted. To assess whether the changes in ceramide levels were indirectly influenced by changes in other clinical variables affected by liraglutide treatment, linear regression models and causal mediation analysis from the R-package “mediation” was applied [44]. Specifically, body weight, HbA1c levels, and urine albumin excretion rate (UAER) was tested for mediation effects.

The LirAlbu12 study was designed as a crossover study, which allowed each participant to be their own control, thus increasing statistical confidence. For this reason ceramide changes of LirAlbu12 were also compared with paired t-test of the endpoints [32]. Correlation between the ceramides and possible confounders were explored with the following variables: HbA_1c_, weight, systolic blood pressure, age, diabetes duration, total cholesterol, LDL cholesterol, triglyceride, UAER, and estimated glomerular filtration rate (eGFR). Pearson correlation coefficients were calculated and the correlations were visualized using the Hmisc [45] and ggcorrplot packages [46]. A two-sided *P*-value < 0.05 was considered statistically significant. The final processing, statistics and visualizations was carried out in R v.4.2.0 [47], the code is available on Github: https://github.com/Asger-W/Liraglutide-Ceramides.

## Results

### Baseline characteristics

The baseline characteristics of the participants are summarized in Table 1 (LirAlbu12) and Table 2 (LiraFlame26). LirAlbu12 included 27 participants with T2D and albuminuria, with a mean (SD) age of 65.3 (7.3) years, diabetes duration of 14.8 (7.1) years and 18.5% (*n* = 5) women. LiraFlame26 involved 102 individuals with T2D randomized to either liraglutide or placebo treatment, with a mean (SD) age of 66.4 (8.2) years, diabetes duration of 13 (8.7) years and 15.7% (*n* = 16) women. At baseline LiraFlame26 participants in the liraglutide group had by chance higher triglyceride levels compared to the placebo group 2.07 (1.19) vs. 1.56 (0.78) mmol/L (*p* = 0.013), but the studies were otherwise balanced. The baseline characteristics of the two trials were overall comparable.

**Table 1:** Baseline characteristics LirAlbu12

	**Start of trial Total**	**Before Liraglutide**	**Before Placebo**	***P*-value**
n	27	27	27	
Age (years)	65.3 (7.3)	65.3 (7.3)	65.3 (7.3)	1
Woman	5 (18.5%)	5 (18.5%)	5 (18.5%)	1
Weight (kg)	99.5 (18.7)	100 (18.5)	99.5 (17.8)	0.923
Diabetes duration (years)	14.8 (7.1)	14.8 (7.1)	14.8 (7.1)	1
HbA_1c_ (mmol/mol)	62 (10.7)	62.6 (11.6)	59.6 (12.1)	0.356
HbA_1c_ (%)	7.8 (1)	7.9 (1.1)	7.6 (1.1)	0.356
Total cholesterol (mmol/L)	3.8 (0.9)	3.8 (1)	3.8 (0.8)	0.886
LDL (mmol/L)	1.88 (0.59)	1.82 (0.50)	1.86 (0.61)	0.768
Triglyceride (mmol/L)	2.20 (1.79)	1.99 (1.31)	2.26 (1.70)	0.518
Systolic blood pressure (mm Hg)	136 (18)	137 (16)	133 (15)	0.355
eGFR (mL/min/1.73m^2^)	75 (23)	75 (23)	73 (23)	0.783
UAER (mg/d)	8 [7-13]	9 [7-14]	8 [7-12]	0.967

Mean (SD), n (%) or median [IQR], groupwise comparison between liraglutide and placebo was carried out using chi square test for categorical variables and ANOVA for continuous variables

*LDL* Low-density lipoprotein, *eGFR* Estimated glomerular filtration rate, *UAER*: Urinary albumin excretion rate

**Table 2:** Baseline characteristics LiraFlame26

	**Total**	**Liraglutide**	**Placebo**	***P*-value**
n	102	51	51	
Age (years)	66.4 (8.2)	65.9 (8.6)	66.9 (7.8)	0.556
Women	16 (15.7%)	6 (11.8%)	10 (19.6%)	0.414
Weight (kg)	91.2 (17.3)	94.5 (19.9)	87.9 (13.6)	0.055
Diabetes duration (years)	13 (8.7)	13.3 (9.1)	12.6 (8.3)	0.657
HbA_1c_ (mmol/mol)	58.4 (10.1)	58.7 (9.6)	58 (10.6)	0.725
HbA_1c_ (%)	7.5 (0.9)	7.5 (0.9)	7.5 (1)	0.725
Total cholesterol (mmol/L)	4.1 (0.8)	4.1 (0.8)	4.1 (0.8)	0.855
LDL (mmol/L)	2.10 (0.67)	2.05 (0.72)	2.15 (0.62)	0.476
Triglyceride (mmol/L)	1.81 (1.03)	2.07 (1.19)	1.56 (0.78)	0.013
Systolic blood pressure (mm Hg)	135 (17)	133 (14)	137 (20)	0.253
eGFR (mL/min/1.73m^2^)	83 (16)	83 (18)	84 (15)	0.746
UAER (mg/d)	2 [2-3]	3 [2-4]	2 [2-3]	0.201

Mean (SD), n (%) or median [IQR], groupwise comparison between liraglutide and placebo was carried out using chi square test for categorical variables and ANOVA for continuous variables

*LDL* Low-density lipoprotein, *Egfr* Estimated glomerular filtration rate, *UAER* Urinary albumin excretion rate

### Ceramides reduced by liraglutide treatment

**Fig. 1:**
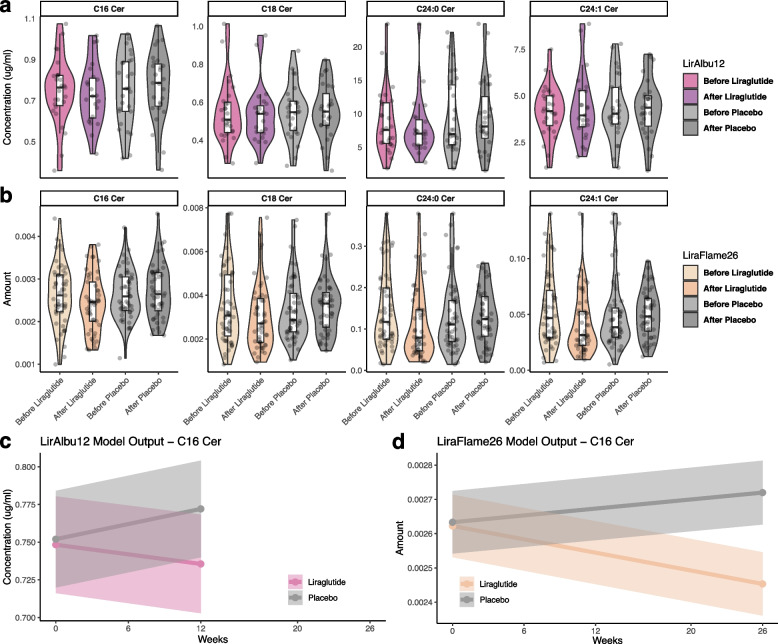
Ceramide distribution at different time points. Boxplots of ceramides before and after liraglutide and placebo in (**A**) LirAlbu12 and (**B**) LiraFlame26. Line graphs of C16 Cer amount per week over the two trials, modeled as linear mixed models: *Ceramide* ~ *Treatment* + *Time* + *Treatment:Time* + *(1|Participant ID),* for (**C**) LirAlbu12 and (**D**) LiraFlame26. The ceramide amount was measured as the ratio of ceramide intensity divided by the intensity of pure standard (peak area of ceramide / peak area ceramide standard)

Changes in ceramide levels were investigated using linear mixed models allowing for random effect between individuals. The ceramide amount was measured as the ratio of ceramide intensity divided by the intensity of pure standard (peak area of ceramide / peak area ceramide standard). At the end of LiraFlame26 the liraglutide group had significantly lower levels of C16 Cer and C24:1 Cer compared to the placebo group. The estimated differences (SD) were -2.556*10^–4^ (1.260*10^–4^) and -1.342*10^–2^ (6.680*10^–3^) for C16 Cer and C24:1 Cer respectively, with *P*-values of 0.045 and 0.047 (Table 3). Specifically, the mean C16 Cer level was 9.5% lower in the liraglutide group after treatment compared to the placebo group, and the C24:1 Cer level was 18.4% lower (Fig. 1). The other investigated ceramides were not significantly altered by liraglutide treatment compared to placebo.

**Table 3:** Ceramide change following liraglutide

	**LirAlbu12**			**LiraFlame26**		
	**Estimated ceramide change**	**Standard Error**	***P*-value**	**Estimated ceramide change**	**Standard Error**	***P*-value**
C16 Cer	-0.033	0.028	0.240	-2.556*10^–4^	1.260*10^–4^	0.045*
C18 Cer	-0.015	0.033	0.639	-6.000*10^–4^	3.459*10^–4^	0.086
C20 Cer	-0.031	0.053	0.559	2.953*10^–5^	2.629*10^–5^	0.264
C22 Cer	-0.239	0.252	0.344	-1.272*10^–4^	1.690*10^–4^	0.454
C24 Cer	-1.006	1.075	0.352	-3.108*10^–2^	1.929*10^–2^	0.110
C24:1 Cer	0.089	0.326	0.786	-1.342*10^–2^	6.680*10^–3^	0.047*

Linear mixed models were constructed for each ceramide in both trials with the following formula:

$$Ceramide\:\sim\:Treatment\:+Time+Treatment:Time+(1\vert Participant\;ID)$$

^*^ Indicate *P*-value < 0.05

The ceramides change of the LirAlbu12 trial were not significantly different between liraglutide and placebo treatment in the linear mixed models (Table 3). Paired t-test of end-to-end differences in the LirAlbu12 trial showed that 12-week liraglutide treatment led to significantly lower levels of C16 Cer (*p* = 0.039) and C24 Cer (*p* = 0.045) compared to placebo (Supplementary Table 1).

### Ceramides in relation to weight and other outcomes

Liraglutide can induce weight loss and lower blood glucose, which have been observed in the clinical trials examined here [32, 33], an investigation was carried out determining if weight loss or lower HbA_1C_ mediated changes to the ceramides. Weight loss did not mediate changes in ceramides levels in any of the trials, with *p* > 0.05 for the average causal mediation effect (ACME) (Table 4). Thus, body weight loss is not a requirement for lowering ceramides with liraglutide. Mediation analysis showed divergent results for HbA1c. The LirAlbu12 trial suggested that changes in HbA1c may be influencing some of the changes seen in C16 Cer, C22 Cer, and C24 Cer, mediation, assessed by ACME, showed *P*-values of 0.034, 0.028, and 0.008, respectively (Table 5). In contrast no indication of mediation for HbA1c was found in the LiraFlame26 trial.

**Table 4:** Change in body weight following liraglutide does not mediate change in ceramide levels

	**LirAlbu12**				**LiraFlame26**			
	**Total effect estimate**	**Total effect *P*-value**	**ACME estimate**	**ACME *P*-value**	**Total effect estimate**	**Total effect *P*-value**	**ACME estimate**	**ACME *P*-value**
**C16 Cer**	0.025 (-0.03;0.079)	0.372	-0.004 (-0.032;0.021)	0.718	2.634*10^–4^ (1.954*10^–5^;5.004*10^–4^)	0.044	7.641*10^–6 ^(-1.421*10^–4^;1.796*10^–4^)	0.908
**C18 Cer**	0.024 (-0.038;0.088)	0.492	0.003 (-0.028;0.034)	0.758	5.527*10^–4^ (-1.182*10^–4^;1.259*10^–3^)	0.102	4.530*10^–5 ^(-3.698*10^–4^;4.983*10^–4^)	0.798
**C20 Cer**	0.037 (-0.053;0.141)	0.45	0.002 (-0.048;0.047)	0.912	-2.777*10^–5^ (-7.603*10^–5^;2.105*10^–5^)	0.314	2.285*10–5 (-1.929*10^–6^;5.199*10^–5^)	0.076
**C22 Cer**	0.281 (-0.21;0.775)	0.262	0.031 (-0.187;0.241)	0.724	1.432*10^–4^ (-1.988*10^–4^;4.755*10^–4^)	0.37	2.973*10^–5 ^(-1.230*10^–4^;2.119*10^–4^)	0.788
**C24 Cer**	1.272 (-0.762;3.59)	0.238	-0.238 (-1.312;0.687)	0.716	2.703*10^–2^ (-9.487*10^–3^;6.170*10^–2^)	0.168	-1.562*10–3 (-2.448*10^–2^;1.971*10^–2^)	0.88
**C24:1 Cer**	-0.025 (-0.687;0.642)	0.958	0.074 (-0.205;0.403)	0.616	1.178*10^–2^ (-2.336*10^–3^;2.387*10^–2^)	0.094	-9.104*10^–4 ^(-8.647*10^–3^;6.183*10^–3^)	0.85

Mediation effect was assessed by linear regression models with and without adjustment for the tested confounder the effect and significance were estimated by bootstrapping 1000 times using the mediator package in R

*ACME *Average casual mediation effect

**Table 5:** Change in HbA1c following liraglutide might mediate change in ceramide levels

	**LirAlbu12**				**LiraFlame26**			
	**Total effect estimate**	**Total effect *P*-value**	**ACME estimate**	**ACME *P*-value**	**Total effect estimate**	**Total effect *P*-value**	**ACME estimate**	**ACME *P*-value**
C16 Cer	0.03 (-0.027;0.083)	0.318	0.039 (0.002;0.084)	0.034	2.634*10^–4^ (1.248*10^–5^;4.898*10^–4^)	0.026	4.980*10^–5^ (-8.159*10^–5^;9.854*10^–5^)	0.298
C18 Cer	0.016 (-0.047;0.073)	0.664	0.054 (-0.037;0.132)	0.274	5.527*10^–4^ (-1.136*10^–4^;1.177*10^–3^)	0.092	2.454*10^–4^ (-1.558*10^–4^;3.916*10^–4^)	0.218
C20 Cer	0.023 (-0.068;0.125)	0.7	0.099 (-0.012;0.205)	0.094	-2.777*10^–5^ (-7.732*10^–5^;2.150*10^–5^)	0.272	2.416*10^–6^ (-7.150*10^–6^;3.659*10^–5^)	0.686
C22 Cer	0.227 (-0.244;0.755)	0.374	0.553 (0.078;1.025)	0.028	1.432*10^–4^ (-1.994*10^–4^;4.657*10^–4^)	0.384	-5.123*10^–5^ (-1.410*10^–4^;2.217*10^–5^)	0.14
C24 Cer	1.031 (-0.971;3.167)	0.32	3.019 (0.465;5.82)	0.008	2.703*10^–2^ (-1.441*10^–2^;6.561*10^–2^)	0.172	1.250*10^–2^ (-1.552*10^–2^;2.195*10^–2^)	0.402
C24:1 Cer	-0.089 (-0.732;0.569)	0.762	0.422 (-0.191;0.988)	0.22	1.178*10^–2^ (-1.764*10^–3^;2.417*10^–2^)	0.084	4.853*10^–3^ (-4.723*10^–3^;8.500*10^–3^)	0.354

Mediation effect was assessed by linear regression models with and without adjustment for the tested confounder the effect and significance were estimated by bootstrapping 1000 times using the mediator package in R

*ACME *Average casual mediation effect

Analysis of correlations between ceramide concentrations and outcome variables were investigated, to better understand their relationship. The ceramides were positively correlated with other lipid measures such as triglycerides and total cholesterol (correlation estimates between 0.19–0.55, *p* < 0.05), but not LDL cholesterol (Supplementary Fig. 1), adjusting the linear mixed models for these lipid measures did not affect the result, except when adjusting for triglycerides, here C16 Cer and C24:1 Cer lost statistical significance in the LiraFlame26 trial (Supplementary Tables 2, 3 and 4) No ceramide showed significant correlation to blood pressure or weight loss.

In the LirAlbu12 trial C20 Cer, C22 Cer and C24 Cer all showed significant correlation to HbA_1c_ with estimates (95% CI) 0.23 (0.042; 0.407), 0.21 (0.02; 0.388), 0.23 (0.035; 0.401) and *p* = 0.017, 0.031, 0.021 respectively. However, no such correlation between ceramides and HbA1c was found in the LiraFlame26 trial.

In the LiraFlame26 trial, C24 Cer and C24:1 Cer show positive correlation and C20 Cer show a negative correlation to UAER. In contrast, all investigated ceramides in the LirAlbu12 trial showed a significant positive correlation to UAER (correlation estimates between 0.22–0.49), one the main outcomes of the LirAlbu12 trial. Previous studies have found associations between kidney disease and ceramides [48, 49], thus mediation of UAER on ceramide change were investigated. Changes in UAER appeared to mediate the changes of C20 Cer, C22 Cer, C24 Cer and C24:1 Cer, but not C16 Cer, mediation, assessed by ACME, resulted in *P*-values of 0.03, 0.016, 0.008, 0.026 and 0.872 respectively in the LirAlbu12 trial. No indication of UAER mediation was found in the LiraFlame26 trial with all ACME *p* > 0.05 (Supplementary Table 5).

## Discussion

Understanding the potential influence of GLP-1RA on ceramides holds promise for improving CVD risk management in people with T2D, this is especially true if ceramides are found to be causative of CVD as has been suggested in a recent review [50]. In that case, a precision medicine approach would be to use GLP1-RA for lowering ceramides and hence CVD risk in the at-risk patients.

This study examined the impact of liraglutide treatment on ceramides that have previously been shown associated with CV risk and all-cause mortality. The results showed that individuals treated with liraglutide had significantly lower levels of C16 Cer and C24:1 Cer compared to placebo treated after 26-weeks of treatment, but not after 12 weeks suggesting liraglutide treatment needs to be sustained for more than 12 weeks to induce significant changes to the ceramide levels. While not all the ceramides investigated reached changes that were statistical significance, there was a consistent trend of decreasing ceramide levels following liraglutide treatment across two independent clinical trials.

### Therapy for ceramide reduction

The findings presented here correspond well with previous studies on the effects of liraglutide and other GLP-1RAs on ceramides [12, 51–54]. One study, on people with obesity (*n* = 32), found that after one year of follow-up, those treated with liraglutide (1.2 mg/d) maintained a stable level of C16 Cer, whereas those without liraglutide treatment had an increase in the level of C16 Cer [52]. Jendle et al. reported a decrease in ceramides after 18 weeks of treatment with liraglutide (1.8 mg/d) and the sulfonylurea glimepiride (4 mg/d) respectively, in individuals with T2D (*n* = 62), ceramides decreased more with liraglutide treatment than with glimepiride [53]. In the phase 2 trial for tirzepatide, a dual-agonist activating both the GLP-1 and the glucose-dependent insulinotropic polypeptide receptors, people (*n* = 314) with T2D were randomized to placebo or tirzepatide treatment for 26 weeks. The levels of C22 Cer and C24 Cer were lower in people treated with tirzepatide (15 mg/w) in comparison to the placebo group [51]. Zhang et al. investigated the GLP-1RA exenatide and reported unchanged ceremide levels for the six ceramides in people (*n* = 35) with T2D after 12 weeks of exenatide treatment (20 µg/d) [12], there are many possible explanations as these are two different drug molecules, and overall, the results were in line with our 12 weeks results. Taken together, these findings suggest that lowering of ceramide is a likely effect of GLP-1RAs treatments, but to a varying extent. Furthermore, several studies reported improvements in lipoprotein profiles following treatment with liraglutide [55–58], indicating that its effects on the lipidome may not be limited to ceramides. The lipid modulating effects of liraglutide and other GLP-1RAs are worth considering when selecting treatments for individuals with T2D, particularly for those with highest CVD risk.

### Ceramide in relation to clinical outcomes

The LiraFlame26 trial showed fewer correlations between ceramides and other risk factors compared to the LirAlbu12 trial. These differences could be because the number of participants was smaller in the LirAlbu12 trial or because of the crossover design, led to lower ceramide variation. Interestingly, weight loss did not mediate the reduction of ceramide levels in these studies. This is in agreement with Akawi et al. who observed suppression of C16 Cer following one year liraglutide treatment (1.2 mg/d) compared to placebo (*n* = 32), but did not observe changes in BMI [52]. Other studies have observed a simultaneous reduction of ceramide levels and weight, but have not investigated, whether the weight loss mediates the reduction in ceramide levels [20, 59, 60].

It is worth noting that, unlike the LiraFlame26 trial, the LirAlbu12 trial had an inclusion criterion on albuminuria. Mediation analysis suggested that some of the observed effect of ceramides reduction in the LirAlbu12 trial could be mediated by a reduction in HbA_1C_ and UAER. The ceramides investigated in the present study have previously been linked to kidney disease. A study by Liu et al. found that C16 Cer levels were significantly higher in people with overt diabetic kidney disease [48]. Similarly, Mantovani et al. found increased levels of all six ceramides, investigated in this paper, in people with chronic kidney disease compared to those without chronic kidney disease [49]. Comparable findings have been made in a mouse model [61]. As lower UAER and lower ceramide levels both are linked to improved kidney health, a correlation between UAER and ceramide levels could be expected.

This study demonstrated that 26-week liraglutide treatment lowered C16 Cer and C24:1 Cer levels in individuals with T2D, compared to placebo, despite the effect of ceramide change was relatively modest and the interindividual differences showed high variation.

### Strengths and limitations

A strength of the present study is that it combines the effects observed in two independent trials one with a crossover and one with a parallel trial design.

A limitation for this post-hoc study is that it was not designed with ceramides as an outcome. More studies are needed to determine if longer treatment with liraglutide can significantly reduce the concentrations of ceramides associated to CVD. Additionally, this study did not control for diet, exercise, or medication changes, all of which could affect ceramide levels.

## Conclusions

Here an investigation was carried out on the impact of liraglutide treatment on ceramides in two clinical trials including individuals with T2D, as the ceramides a known to be associated with CVD risk. C16 Cer and C24:1 Cer was significantly reduced following 26-weeks liraglutide treatment compared to placebo and that more than 12-weeks of treatment is needed to achieve significant ceramide lowering. These results suggest that liraglutide treatment can be used as a ceramide lowering intervention which could potentially have significant implications in precision medicine initiatives for CV risk. However, further research is needed to determine if lowering of ceramides translates to decreased CV risk.

### Supplementary Information

**Additional file 1: Supplementary Figure 1.** Ceramide correlation matrix. **Supplementary Table 1.** Ceramide measures by treatment group in LirAlbu12. **Supplementary Table 2.** Ceramide change following liraglutide adjusted for LDL. **Supplementary Table 3.** Ceramide change following liraglutide adjusted for total cholesterol. **Supplementary Table 4.** Ceramide change following liraglutide adjusted for total triglyceride. **Supplementary Table 5.** Change in UAER following liraglutide might mediate change in ceramide levels.

## Data Availability

The dataset analyzed here is not publicly available, for the privacy of the participants, in compliance with EU and Danish data protection law. The data can be accessed upon reasonable request; relevant legal permission from the data protection agency is required. Data access request should be directed to PR, peter.rossing@regionh.dk. The code used for data analysis is available on github: https://github.com/Asger-W/Liraglutide-Ceramides.

## Abbreviations

ADE: Average direct effect
ACME: Average causal mediation effect
Cer: Ceramide
CVD: Cardiovascular disease
C16 Cer: Ceramide(d18:1/16:0)
C18 Cer: Ceramide(d18:1/18:0)
C17 Cer: Ceramide(d18:1/17:0)
C20 Cer: Ceramide(d18:1/20:0)
C22 Cer: Ceramide(d18:1/22:0)
C24 Cer: Ceramide(d18:1/24:0)
C24:1 Cer: Ceramide(d18:1/24:1)
eGFR: Estimated glomerular filtration rate
HbA1c: Hemoglobin A1C
LDL: Low-density lipids
RA: Receptor agonist
SCD1: Stearoyl-CoA desaturase-1
T2D: Type 2 Diabetes
UAER: Urinary albumin excretion rate
