# Supplementary material for: Ceramides are decreased after liraglutide treatment in people with type 2 diabetes: a post hoc analysis of two randomized clinical trials
Source: Lipids Health Dis. 2023 Sep 26;22:160. doi: 10.1186/s12944-023-01922-z (PMC10521385; doi:10.1186/s12944-023-01922-z)
Supplement: Supplementary file 1 — Additional file 1: Supplementary Figure 1. Ceramide correlation matrix. Supplementary Table 1. Ceramide measures by treatment group in LirAlbu12. Supplementary Table 2. Ceramide change following liraglutide adjusted for LDL. Supplementary Table 3. Ceramide change following liraglutide adjusted for total cholesterol. Supplementary Table 4. Ceramide change following liraglutide adjusted for total triglyceride. Supplementary Table 5. Change in UAER following liraglutide might mediate change in ceramide levels. [file 12944_2023_1922_MOESM1_ESM.docx]

**Supplementary Figure 1 – Ceramide correlation matrix**


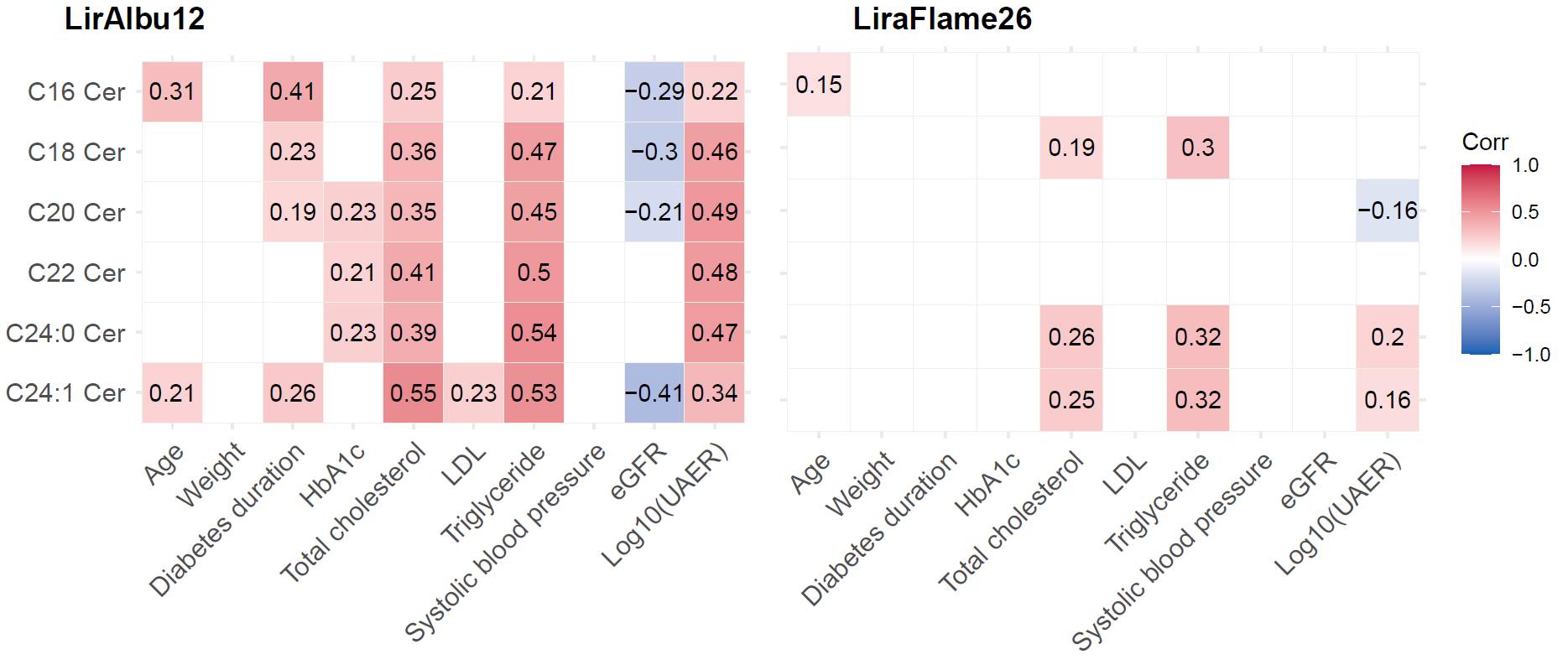


Pearson’s correlation between ceramides and possible confounders, numbers indicate estimates, only correlation with a p-value < 0.05 is shown. LDL: Low-density lipoprotein, eGFR: Estimated glomerular filtration rate, UAER: Urinary albumin excretion rate.

**Supplementary Table 1 – Ceramide measures by treatment group in LirAlbu12**

|  | Before  liraglutide | After  liraglutide | Change liraglutide  (95% CI) | Before placebo | After placebo | Change placebo  (95% CI) | End vs. end liraglutide compared to  placebo (95% CI) |
| --- | --- | --- | --- | --- | --- | --- | --- |
| C16 Cer | 0.748 (0.162) | 0.732 (0.149) | -0.010 (-0.056; 0.036), P=0.665 | 0.752  (0.171) | 0.772 (0.167) | 0.020 (-0.016; 0.056), P=0.266 | -0.039 (-0.075;  -0.002), P = 0.039* |
| C18 Cer | 0.546 (0.164) | 0.534 (0.153) | -0.012 (-0.068; 0.044), P=0.657 | 0.550  (0.141) | 0.553 (0.142) | 0.004 (-0.030; 0.037), P=0.832 | -0.019 (-0.071;  .033), P = 0.455 |
| C20 Cer | 0.644 (0.230) | 0.620 (0.227) | -0.021 (-0.106; 0.063), P=0.604 | 0.665  (0.239) | 0.667 (0.264) | 0.001 (-0.062; 0.064), P=0.969 | -0.046 (-0.142;  0.05), P = 0.333 |
| C22 Cer | 2.620 (1.090) | 2.478 (1.182) | -0.194 (-0.607; 0.219), P=0.341 | 2.797 (1.246) | 2.829 (1.357) | 0.033 (-0.292; 0.357), P=0.837 | -0.381 (-0.81;  0.048), P =0.079 |
| C24 Cer | 8.773 (4.824) | 7.984  (4.329) | -1.152 (-3.098; 0.794), P=0.233 | 9.620 (5.660) | 9.499 (5.445) | -0.121 (-1.435; 1.194), P=0.852 | -1.729 (-3.418;  -0.04), P = 0.045* |
| C24:1 Cer | 4.156 (1.360) | 4.373  (1.829) | 0.076 (-0.488; 0.640), P=0.782 | 4.357 (1.699) | 4.344 (1.557) | -0.013 (-0.380; 0.355), P=0.944 | -0.098 (-0.705;  0.509, P = 0.741 |

Mean (SD) ceramide amount in µg/ml, * indicate p-value <0.05. Mean change from start to end were tested using 2-sample t-tests. End vs end differences in ceramide concentration between liraglutide (1.8 mg/d) and placebo were assessed with paired t-test.

**Supplementary Table 2 – Ceramide change following liraglutide adjusted for LDL**

|  |  | **LirAlbu12** |  |  |  | **LiraFlame26** |  |
| --- | --- | --- | --- | --- | --- | --- | --- |
|  | **Estimated ceramide change** | **Standard Error** | ***P*-value** |  | **Estimated ceramide change** | **Standard Error** | ***P*-value** |
| C16 Cer | -0.013 | 0.024 | 0.591 |  | -2.81*10^-4^ | 1.26*10^-4^ | 0.028* |
| C18 Cer | -0.006 | 0.023 | 0.808 |  | -6.10*10^-4^ | 3.35*10^-4^ | 0.072 |
| C20 Cer | -0.031 | 0.045 | 0.492 |  | 2.85*10^-5^ | 2.68*10^-5^ | 0.290 |
| C22 Cer | -0.236 | 0.222 | 0.292 |  | -1.78*10^-4^ | 1.76*10^-4^ | 0.315 |
| C24 Cer | -0.656 | 0.919 | 0.478 |  | -3.72*10^-2^ | 1.89*10^-2^ | 0.053 |
| C24:1 Cer | 0.012 | 0.267 | 0.965 |  | -1.44*10^-2^ | 6.67*10^-3^ | 0.033* |

Linear mixed models were constructed for each ceramide in both trials with the following formula:

*Ceramide ~ Treatment+Time + Treatment:Time + LDL + (1|Participant ID).* * Indicate *P*-value <0.05.

**Supplementary Table 3 – Ceramide change following liraglutide adjusted for total cholesterol**

|  |  | **LirAlbu12** |  |  |  | **LiraFlame26** |  |
| --- | --- | --- | --- | --- | --- | --- | --- |
|  | **Estimated ceramide change** | **Standard Error** | ***P*-value** |  | **Estimated ceramide change** | **Standard Error** | ***P*-value** |
| C16 Cer | -0.015 | 0.026 | 0.561 |  | -2.751*10^-4^ | 1.205*10^-4^ | 0.025* |
| C18 Cer | 0.018 | 0.027 | 0.505 |  | -6.562*10^-4^ | 3.267*10^-4^ | 0.047* |
| C20 Cer | 0.021 | 0.043 | 0.629 |  | 3.016*10^-5^ | 2.640*10^-5^ | 0.256 |
| C22 Cer | -0.008 | 0.216 | 0.972 |  | -1.228*10^-4^ | 1.692*10^-4^ | 0.470 |
| C24 Cer | 0.041 | 0.924 | 0.964 |  | -3.540*10^-2^ | 1.757*10^-2^ | 0.047* |
| C24:1 Cer | 0.426 | 0.269 | 0.117 |  | -1.475*10^-2^ | 6.275*10^-3^ | 0.021* |

Linear mixed models were constructed for each ceramide in both trials with the following formula:

*Ceramide ~ Treatment+Time + Treatment:Time + Cholesterol + (1|Participant ID).* * Indicate *P*-value <0.05.

**Supplementary Table 4 – Ceramide change following liraglutide adjusted for total triglyceride**

|  |  | **LirAlbu12** |  |  |  | **LiraFlame26** |  |
| --- | --- | --- | --- | --- | --- | --- | --- |
|  | **Estimated ceramide change** | **Standard Error** | ***P*-value** |  | **Estimated ceramide change** | **Standard Error** | ***P*-value** |
| C16 Cer | -0.035 | 0.026 | 0.173 |  | -2.354*10^-4^ | 1.253*10^-4^ | 0.063 |
| C18 Cer | -0.008 | 0.026 | 0.761 |  | -4.887*10^-4^ | 3.366*10^-4^ | 0.150 |
| C20 Cer | -0.026 | 0.045 | 0.564 |  | 3.024*10^-5^ | 2.629*10^-5^ | 0.253 |
| C22 Cer | -0.239 | 0.215 | 0.270 |  | -1.514*10^-4^ | 1.671*10^-4^ | 0.367 |
| C24 Cer | -0.896 | 0.838 | 0.289 |  | -2.461*10^-2^ | 1.836*10^-2^ | 0.183 |
| C24:1 Cer | 0.088 | 0.263 | 0.739 |  | -1.131*10^-2^ | 6.431*10^-3^ | 0.082 |

Linear mixed models were constructed for each ceramide in both trials with the following formula:

*Ceramide ~ Treatment+Time + Treatment:Time + Triglycerides + (1|Participant ID).* * Indicate *P*-value <0.05.

**Supplementary Table 5 – Change in UAER following liraglutide might mediate change in ceramide levels**

|  | LirAlbu12 | | | |  | LiraFlame26 | | | | |
| --- | --- | --- | --- | --- | --- | --- | --- | --- | --- | --- |
|  | **Total effect estimate** | **Total effect *P*-value** | **ACME estimate** | **ACME *P*-value** |  | **Total effect estimate** | **Total effect *P*-value** | **ACME estimate** | **ACME *P*-value** | **Total effect estimate** |
| C16 Cer | 0.03  (-0.024;0.085) | 0.304 | 0.002  (-0.022;0.031) | 0.872 |  | 2.883*10^-4^ (4.133*10^-5^ ;5.397*10^-4^) | 0.02 | -4.061*10^-5^ (-1.156*10^-4^  ;3.403*10^-5^) | 0.264 | 2.883*10^-4^ (4.133*10^-5^ ;5.397*10^-4^) |
| C18 Cer | 0.016  (-0.051;0.081) | 0.612 | 0.01  (-0.009;0.043) | 0.35 |  | 6.447*10^-4^  (-5.754*10^-5^ ;1.316*10^-3^) | 0.076 | -8.054*10^-5^  (-3.151*10^-4^ ;7.562*10^-5^) | 0.332 | 6.447*10^-4^  (-5.754*10^-5^ ;1.316*10^-3^) |
| C20 Cer | 0.024  (-0.072;0.133) | 0.65 | 0.047  (0.003;0.127) | 0.03 |  | -3.459*10^-5^ (-9.307*10^-5^ ;1.941*10^-5^) | 0.234 | 1.876*10^-5^  (-1.824*10^-6^ ;4.053*10^-5^) | 0.066 | -3.459*10^-5^ (-9.307*10^-5^ ;1.941*10^-5^) |
| C22 Cer | 0.241  (-0.264;0.74) | 0.346 | 0.286  (0.035;0.688) | 0.016 |  | 1.308*10^-4^  (-2.038*10^-4^ ;4.675*10^-4^) | 0.428 | 8.857*10^-6^  (-6.075*10^-5^ ;1.160*10^-4^) | 0.784 | 1.308*10^-4^  (-2.038*10^-4^ ;4.675*10^-4^) |
| C24 Cer | 1.189  (-0.891;3.277) | 0.286 | 1.272  (0.236;2.673) | 0.008 |  | 3.075*10^-2^  (-6.074*10^-3^ ;6.806*10^-2^) | 0.096 | -3.568*10^-3^ (-1.669*10^-2^ ;4.336*10^-3^) | 0.346 | 3.075*10^-2^  (-6.074*10^-3^ ;6.806*10^-2^) |
| C24:1 Cer | -0.069  (-0.675;0.558) | 0.828 | 0.266  (0.022;0.739) | 0.026 |  | 1.357*10^-2^  (-2.218*10^-4^ ;2.626*10^-2^) | 0.056 | -8.651*10^-4^ (-4.897*10^-3^ ;2.050*10^-3^) | 0.558 | 1.357*10^-2^  (-2.218*10^-4^ ;2.626*10^-2^) |

Mediation effect was assessed by linear regression models with and without adjustment for the tested confounder the effect and significance were estimated by bootstrapping 1000 times using the mediator package in R. ACME: Average casual mediation effect. UAER: Urinary albumin excretion rate.
